# Supplementary material for: Identification of Antisense RNA NRAS-AS and Its Preliminary Exploration of the Anticancer Regulatory Mechanism
Source: Genes (Basel). 2024 Nov 27;15(12):1524. doi: 10.3390/genes15121524 (PMC11675080; doi:10.3390/genes15121524)
Supplement: Supplementary file 1 [file genes-15-01524-s001.zip › Figure S1.pdf]

Figure S1 The chromosome location, MFE and Secondary structure prediction of NRAS-AS

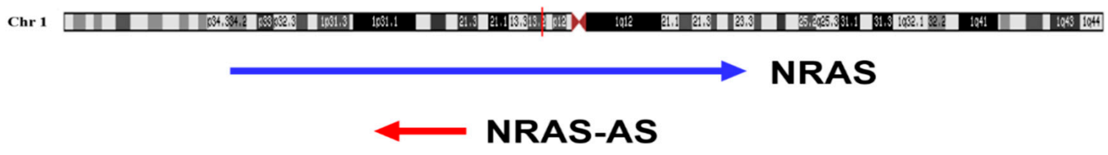

The chromosome location of NRAS-AS and its relationship with NRAS

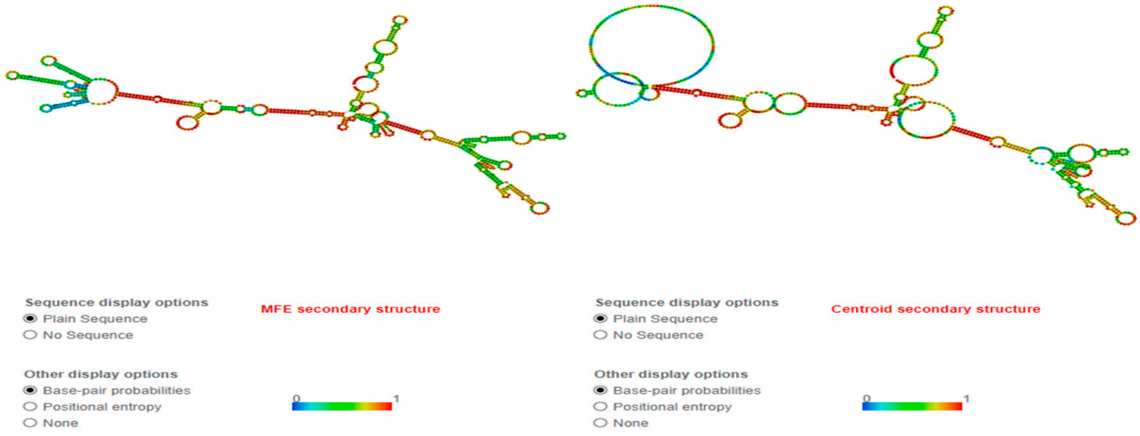

The MFE and Secondary structure prediction of NRAS-AS
