# Supplementary material for: Identification of Antisense RNA NRAS-AS and Its Preliminary Exploration of the Anticancer Regulatory Mechanism
Source: Genes (Basel). 2024 Nov 27;15(12):1524. doi: 10.3390/genes15121524 (PMC11675080; doi:10.3390/genes15121524)
Supplement: Supplementary file 1 [file genes-15-01524-s001.zip › Table S1.pdf]

Table S1：

Information on RT-qPCR primer sequences and reaction procedures for NRAS and NRAS-AS

Sequence information of NRAS and NRAS-AS chain-specific primers

Gene	Primer	Primer sequence
NRAS-AS	RT-Primer	5'-GTTATCGGCTCTATTCTC-3'
	Forward primer	5'-TCAGTGGAATAGATGTCTCA-3'
	Reverse primer	5'-AATGGCATCTGCTCTCAA-3'
NRAS	RT-Primer	5'-ATGACTGAGGTGATAAGC-3'
	Forward primer	5'-GCCACTTTGTTCCTGTCT-3'
	Reverse primer	5'-TTAGCAGTAAGAAGCACAAC-3'

RT-qPCR reaction procedure

Steps	Temperature	Time	Cycles
predenaturation	95° C	2min	
denaturation	95° C	30sec	35cycle
annealing	68° C	30sec	
extension	72° C	1min	
extension	72° C	10min	
save	4° C		
