# Supplementary material for: Identification of Antisense RNA NRAS-AS and Its Preliminary Exploration of the Anticancer Regulatory Mechanism
Source: Genes (Basel). 2024 Nov 27;15(12):1524. doi: 10.3390/genes15121524 (PMC11675080; doi:10.3390/genes15121524)
Supplement: Supplementary file 1 [file genes-15-01524-s001.zip › Table S2.pdf]

Table S2:

The amplification of the 5' and 3' ends of the NRAS-AS transcript, the following primers were used:

Primer	Primer sequence
5’RACE Primer A	CAGGAGCGGATCAAGGCG GAGAGGAAG
3’RACE Primer A	TGGATACCCTTGGCTTTAGTTCTCGGACAC
UPM Long primer	5′-CTAATACGACTCACTATAGGGCAAGCAGTGGTAT CAACGCAGAGT-3′
UPM Short primer	5′-CTAATACGACTCACTATAGGGC- 3′
SMARTer II A Oligonucleotide	5'-AAGCAGTGGTATCAACGCAGAGTACATGGG-3′

The amplification was performed using the following PCR conditions:

Steps	Temperature	Time	Cycles
Initial denaturation	94℃	5min	
Denaturation	94℃	30sec	35 cycles
Annealing	68℃	30sec	
Extension	72℃	3min	
Final extension	72℃	7min	
Storage	4℃		
